# Small intestinal gastrointestinal stromal tumor in a young adult woman: a case report and review of the literature

**DOI:** 10.1186/1752-1947-8-321

**Published:** 2014-09-28

**Authors:** Suzana Manxhuka-Kerliu, Vjollca Sahatciu-Meka, Irma Kerliu, Argjira Juniku-Shkololli, Lloreta Kerliu, Mevlyde Kastrati, Vesa Kotorri

**Affiliations:** 1 Institute of Pathology, Faculty of Medicine, University of Prishtina, Mother Theresa Street NN, 10 000 Prishtina, Kosovo; 2 Faculty of Medicine, University of Prishtina, Mother Theresa Street NN, 10 000 Prishtina, Kosovo; 3 Massachusetts College of Pharmacy and Health Sciences (MCPHS), 179 Longwood Avenue, Boston, MA 02115, USA; 4 Gastroenterology Clinic, University Clinical Center of Kosovo, Mother Theresa Street NN, 10 000 Prishtina, Kosovo

**Keywords:** Gastrointestinal stromal tumors (GIST) in young adults, Morphology, Immunohistochemistry

## Abstract

**Introduction:**

Gastrointestinal stromal tumor is the most common sarcoma of the gastrointestinal tract. We report a case of gastrointestinal stromal tumor in a small intestine, initially suspected for leiomyosarcoma given that gastrointestinal stromal tumors in young adult patients are limited due to their rarity.

**Case presentation:**

A 30-year-old Caucasian ethnic Albanian woman from Kosovo presented with abdominal pain, nausea and vomiting. Subsequently, the tumor was detected in her small intestine, as an infiltrating mass approximately 10cm in diameter. The tumor was resected en bloc and duodenojejunal terminal-terminal anastomosis was performed. The tumor was a large, bulky, intramural mass, with fish-flesh to tan-brown appearance, as well as with foci of hemorrhage and necrosis. On histological examination the tumor showed transmural growth, deep infiltrative pattern and malignant feature, with mitotic count >5 per 50 high-power field, dense cellularity with plump spindle cells, and with eosinophilic cytoplasm within variably hyalinized and edematous stroma, skeinoid fibers (extracellular collagen globules) and foci of hemorrhage. In addition, the tumor was composed of areas with epithelioid morphology. The immunohistochemistry results showed high expression of proto-oncogene c-kit, CD117, CD34 and vimentin, whereas α-smooth muscle actin was focally positive. Desmin and S-100 protein were negative.

**Conclusions:**

Gastrointestinal stromal tumor should be included in the differential diagnoses of intestinal mesenchymal tumors presenting as a single mass in young female adults. Given that gastrointestinal stromal tumors in young adults represent a more heterogeneous group than gastrointestinal stromal tumor in pediatric cases, more effort should be made to investigate its pathogenesis and potentially more specific treatment.

## Introduction

Gastrointestinal stromal tumors (GISTs) are the most common mesenchymal tumors of the gastrointestinal tract. GISTs originate from the interstitial cells of Cajal (ICCs), which are dependent on stem cell factor receptor interaction. Like GISTs, these cells express both KIT and CD34 [1].

The ICCs form a complex cell network within the gastrointestinal tract wall where they function as a pacemaker system. Expression of the c-kit proto-oncogene is essential for the development of this system [2].

Both benign and malignant GISTs commonly show losses in chromosomes 14 and 22 in cytogenetic studies and by comparative genomic hybridization. Losses in 1p and chromosome 15 have been shown less frequently. Gains and high level amplifications occur in malignant GISTs in 3q, 8q, 5p and Xp. A proportion of GISTs, more commonly the malignant examples, show mutations in the regulatory juxtamembrane domain (exon 11) of the c-*kit* gene. These c-*kit* mutations have been shown to represent gain-of-function mutations leading to ligand-independent activation (autophosphorylation) of the tyrosine kinase and further the phosphorylation cascade that leads to mitogenic activation [3].

Riccardo Ricci *et al.* represented the historical growth in genotype and phenotype evidence on GIST since 1998 in its increasing complexity by building a graph, named “GISTogram”, that visually conveys most of the features characterizing GISTs and the probability for each of them, either alone or in combination, to be observed in a single GIST case [4].

Key elements of the consensus, as described here, are the defining role of KIT immunopositivity in the diagnosis of GIST. A proposed scheme for estimating metastatic risk in these lesions, based on tumor size and mitotic count, recognizes that it is probably unwise to use the definitive term “benign” for any GIST, at least at the present time [5].

Given that KIT is immunohistochemically negative in a minority of GISTs, especially in platelet-derived growth factor receptor alpha (*PDGFRA*) gene mutation-harboring GISTs, mutational analyses of c-*kit* and *PDGFRA* genes may be required for a definitive diagnosis of such GISTs [6].

*KIT* mutations in GIST are clustered in four exons. Most common are exon 11 (juxtamembrane domain) mutations that include deletions, point mutations (affecting a few codons), and duplications (mostly in the 3′ region). Exon 9 mutations (5 to 10%) usually are 2-codon 502-503 duplications, and these occur predominantly in intestinal versus gastric GISTs. Most mutations are somatic (in tumor tissue only), however, patients with familial GIST syndrome have constitutional *KIT*/*PDGFRA* mutations; greater than 10 families have been reported worldwide with mutations generally similar to those in sporadic GISTs [7].

For completely resected primary GISTs, mitotic rate, tumor size, and tumor location are important risk factors for recurrence. However, molecular markers for recurrence are still lacking. In the immunohistochemically validated cohort, aurora kinase A (AURKA) expression was significantly higher in nongastric tumors than in gastric tumors and was significantly correlated with Armed Forces Institute of Pathology-Miettinen risk group. By integrating bioinformatics and clinicopathological studies, AURKA was identified as a marker for high-risk GIST [8].

GISTs are a paradigm for the development of personalized treatment for patients with cancer. The study of drug-resistant tumors has advanced our understanding of kinase biology, enabling the development of novel kinase inhibitors. Not all CD117-positive GISTs harbor a *KIT* mutation and vice versa. With the current knowledge, we know that the *KIT*-mutated GIST group is not homogenous in terms of prognosis and tyrosine kinase inhibitor sensitivity, depending on the specific mutation site within the *KIT* gene. Further improvements in GIST treatment may require targeting GIST stem cell populations and/or additional genomic events [9].

Current knowledge demonstrates that the presence of kinase mutations in c-*kit* and *PDGFRA* and their localization within the gene sequence as well as the mutation type are of great importance for planning appropriate treatment [10].

Several studies have shown that response to imatinib in patients with GIST mainly depends on the mutational status of *KIT* or *PDGFRA*. Moreover, most if not all patients treated with imatinib for advanced GIST will secondarily develop progressive disease under treatment. In the majority of cases, such progressions are the result of acquired resistance due to occurrence of secondary c-*kit* mutations, especially for GIST with primary exon 11 mutations. Sunitinib is another approved drug and an inhibitor of multiple tyrosine kinases including KIT, PDGFRA as well as platelet-derived growth factor receptor beta and vascular endothelial growth factor receptors which are associated with angiogenesis [11-14].

Sunitinib treatment may be one of the most important therapeutic options for unresectable imatinib-resistant GIST [15-17].

The clinical activity of sunitinib, after the failure of imatinib, is significantly influenced by both primary and secondary mutations in the predominant pathogenic kinases [18].

Most patients with GIST eventually develop clinical resistance to imatinib and sunitinib. Imatinib and sunitinib resistance generally result from secondary mutations in the KIT and/or PDGFRA kinase domains. Preclinical studies suggest that imatinib- and sunitinib-resistant mutations can be treated using more potent kinase inhibitors, such as nilotinib, which inactivate the mutant kinase proteins. Alternatively, the mutant kinase proteins can be targeted using heat shock protein 90 inhibitors, which result in degradation of activated *KIT* and/or *PDGFRA*, or using KIT transcriptional repressors, such as flavopiridol [19].

In this case report we present a patient with GIST in her small intestine, initially suspected for leiomyosarcoma, given that GISTs in young patients (under 40 years) are limited due to their rarity.

## Case presentation

A 30-year-old Caucasian ethnic Albanian woman from Kosovo presented with abdominal pain, nausea and vomiting. Subsequently, a tumor was detected in her small intestine (duodenojejunum), as an infiltrating mass approximately 10cm in diameter that had infiltrated her pancreatic capsule and radix mesentery. The tumor was resected en bloc and a duodenojejunal terminal-terminal anastomosis was performed. On histological examination, differential diagnoses considered were leiomyosarcoma versus GIST. Immunohistochemistry confirmed the diagnosis of malignant GIST.

### Macroscopic features

The tumor was large, bulky, 10cm in diameter, with tan-brown appearance, as well as with massive hemorrhagic necrosis and cyst formation.

### Histological and phenotypic findings

**Figure 1:**
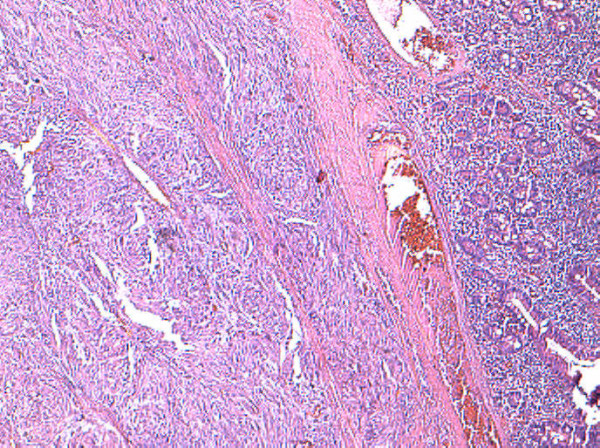
**Intestinal submucosal gastrointestinal stromal tumor with dense cellularity, short fascicles and whorls.** Hematoxylin and eosin stain, 4×.

**Figure 2:**
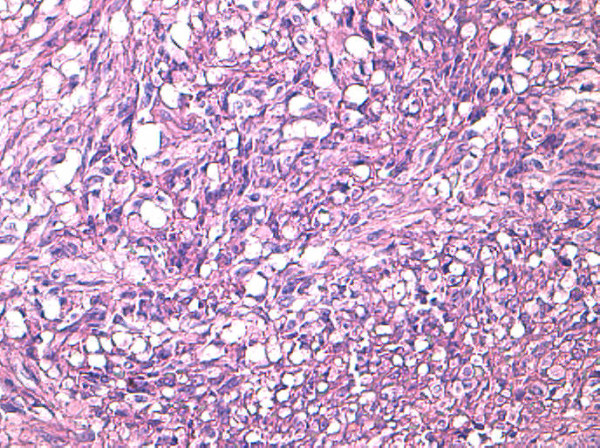
**Epithelioid differentiation with perinuclear and cytoplasmic vacuolization.** Hematoxylin and eosin stain, 20×.

**Figure 3:**
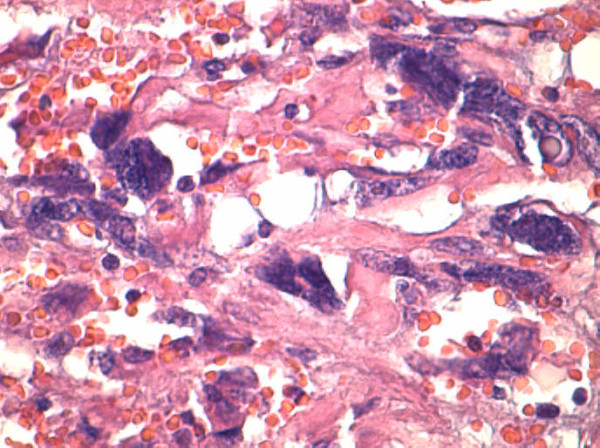
**Spindle cell area of gastrointestinal stromal tumor with nuclear pleomorphism.** Hematoxylin and eosin stain, 40×.

**Figure 4:**
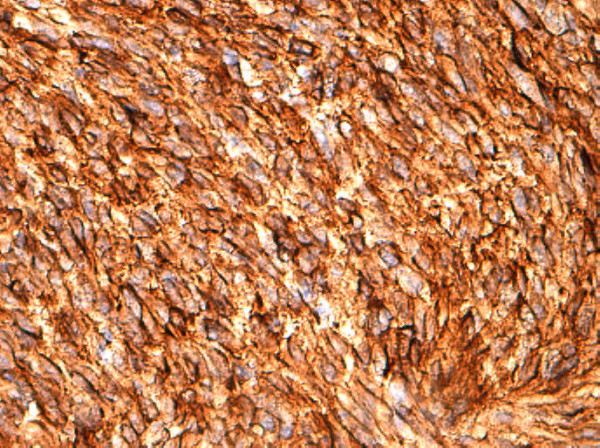
**CD117**^**+ **^**(proto-oncogene c-Kit) strong and diffuse cytoplasmic staining, 20×.**

**Figure 5:**
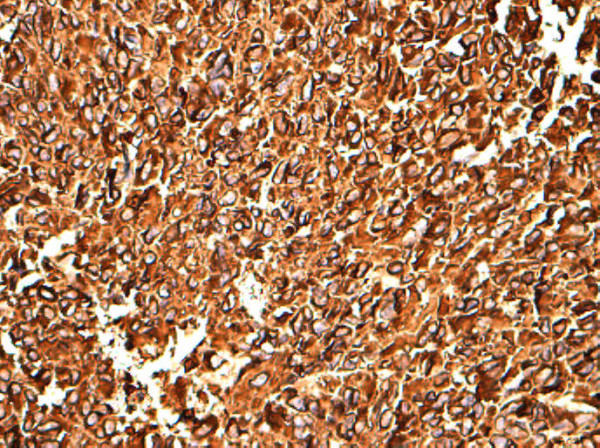
**Vimentin**^**+**^**, strong immunoreactivity, 20×.**

**Figure 6:**
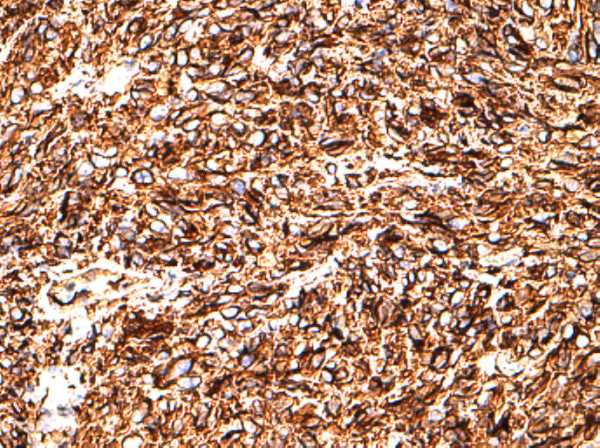
**CD34**^**+ **^**strong and diffuse membrane staining, 20×.**

**Figure 7:**
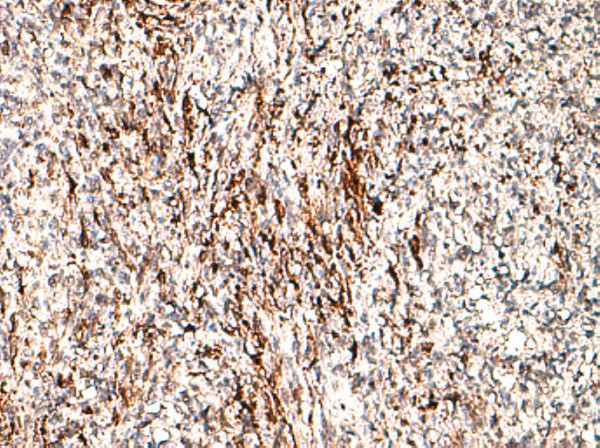
**Smooth muscle actin**^**- **^**focal immunoreactivity of some smooth muscle differentiated cells, 10×.**

**Figure 8:**
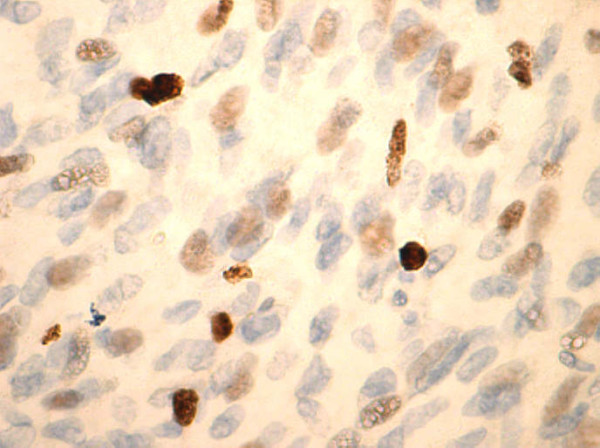
**Ki-67 low proliferation index (10%), 40×.**

On histological examination, the tumor showed transmural growth, deep infiltrative pattern and malignant feature; it was high risk according to Fletcher’s criteria with mitotic count >5 per 50 high-power field (HPF), dense cellularity with plump spindle cells with eosinophilic cytoplasm within variably hyalinized edematous stroma, skeinoid fibers (extracellular collagen globules) and foci of hemorrhage and necrosis. In addition, the tumor was composed of areas with epithelioid morphology (Figures 1, 2 and 3). The immunohistochemistry results showed high expression of proto-oncogene c-Kit (CD117), CD34 and vimentin, whereas α-smooth muscle actin was focally positive. Desmin and S-100 protein were negative. Ki-67 expression showed low proliferative index (10%), (Figures 4, 5, 6, 7 and 8).

Our final diagnosis was intestine GIST, with mixed spindle and epithelioid morphology.

## Discussion

Rare cancer issues including GIST have not yet been explored in depth because of their low incidence. As a result, epidemiological studies are unsuccessful in identifying indisputable etiological risk factors [20-23].

GISTs are the most common tumors among gastrointestinal mesenchymal. GISTs are different from neurogenic tumors, leiomyomas and leiomyosarcomas in initial symptom, tumor location, biological behavior and immunophenotype. Immunohistochemistry plays an important role in differentiating GISTs from leiomyomas and neurogenic tumors.

The origin of Cajal cells and smooth muscle from a common precursor cell, the hybrid of these two seen in many GISTs, and the occurrence of GISTs in the omentum and mesentery, suggest that cells from such origin will more likely differentiate toward a Cajal cell phenotype. Electron microscopic observations showing hybrid autonomic nerve and smooth muscle features in many GISTs are also consistent with origin from a multipotential precursor cell [24].

The resemblance of histomorphological features of our case to that of smooth muscle tumors as well as the patient’s age led us to suspect leiomyosarcoma.

However, immunohistochemistry is conclusive in determining the histology of the tumor, based on proto-oncogene c-kit (CD117) and CD 34 positivity, resulting in the final diagnosis of small intestinal malignant GIST.

Folgado *et al.* found *KIT* mutations in 50% of all 10 patients evaluated. Most cases had a poor prognosis either according to Fletcher’s criteria or given the presence of metastasis [25].

There is a publication based on the investigation of 82 males and 31 females, with a median age of 51 years. The primary sites of GIST were small intestine, stomach and others. The tumor diameter varied from 1 to 26cm. Most common histology was spindle cell morphology followed by mixed spindle cell and epithelioid morphology. No statistically significant association was noted between high risk (HR) Fletcher score, proliferation index- Mib score >10, tumor size >10cm, and the risk of recurrence [26].

A more favorable prognosis in duodenal GISTs is attributed to a smaller size of the lesion, and a low mitotic count [27].

Lv *et al.* consider that tumor sites and total number of microscopic indicators are independent risk factors associated with the prognosis of GIST [28].

Similarly, our case of small intestine GIST shows combined spindle cell and epithelioid morphology, tumor size >10cm, HR Fletcher score, Ki-67 around 10% and without synchronous metastasis.

Anaplastic GIST, with pleomorphic cells and loss of CD117, until recently have only been reported in patients with chronic imatinib mesylate treatment. Dedifferentiated GISTs arising *de novo* is a newly identified entity that may prove to be difficult to diagnose. It was presented in the case of a 52-year-old woman found to have a dedifferentiated GIST without prior imatinib mesylate therapy. This case is the first reported dedifferentiated GIST arising *de novo* from the small bowel, and at 30cm in greatest diameter, the largest reported to date [29].

GISTs are mesenchymal tumors of the intestinal tract that typically occur in adults over the age of 40 years. The occurrence of c-*kit* mutations correlated with the age of patients [30].

GISTs in younger (under 40 years) patients are rare and not well characterized. Of the 10 GISTs in young adults, half occurred in the small bowel and had spindle cell morphology, and one case had lymph node metastasis. *KIT* mutations were identified in seven cases, four in exon 11 and three in exon 9. GISTs that occur in children are a separate clinicopathologic and molecular subset with predilection for girls, multifocal gastric tumors, and wild-type *KIT*/*PDGFRA* genotype. In contrast, GISTs in young adults are a more heterogeneous group, including cases that resemble either the pediatric or the older adult-type tumors [31].

Likewise, we present a young woman (under the age of 40 years) with small intestinal GIST, with c-*kit*’ mutation as well as CD34 overexpression; it shows heterogeneous morphology.

Studies on GISTs in young patients are limited due to their rarity, and none have been conducted in Asian populations. GISTs from patients under the age of 30 years were retrospectively reviewed and were analyzed for clinicopathologic features, immunohistochemistry and mutations for exon 9, 11, 13, and 17 of *KIT* gene and exon 12, 14, and 18 of *PDGFRA* gene. Two pediatric (<18-years old) and 20 young adult (18- to 30-years old) cases of GIST were found. Of the 20 GISTs in young adults, 12 (60%) were from extra-gastric locations (six duodenum, five jejunum, and one esophagus), and 16 (80%) showed a spindle cell morphology. Mutations of *KIT* or *PDGFRA* genes were identified in 14 (78%) of the 18 cases. Compared with cases of pediatric GIST, cases of young adults with GIST are heterogeneous and share the characteristics of both GISTS in pediatric and adult cases [32].

On morphological examination, our case shares characteristics of GISTs with both pediatric and adult cases, spindle cell and epithelioid histology.

Shimomura *et al*. published a case of an 18-year-old girl who presented with abdominal pain; a tumor was subsequently detected in her jejunum. GISTs are rare in pediatric populations and pediatric GISTs occur predominantly in females and are characterized by a multifocal gastric location and a wild-type phenotype for the c-*kit* genes. The features of pediatric GISTs of the small intestine have not yet been categorized and, to date, only 11 cases in patients younger than 18 years have been reported. GISTs of the small intestine were expected to show a better response to imatinib treatment than gastric GISTs because of the alterations in the c-*kit* gene [33].

Correspondingly, our case even though not in the pediatric population but in young adulthood, arises in a female patient as a solitary tumor mass.

Peritoneal and hepatic metastases are the main routes of spread of GIST. However, criteria to predict the site and pattern of recurrence in individual cases are still lacking.

Agaimy *et al*. retrospectively analyzed 67 consecutive GISTs with complete gross descriptions to correlate macroscopic patterns with clinical course. Primary endpoint was the appearance of synchronous or metachronous peritoneal disease. Type I tumors were predominantly gastric and frequently had very low/low risk whereas type II tumors were predominantly intestinal and often of intermediate/high risk. The careful gross and microscopic assessment of a resection specimen harboring GIST is of great importance because it allows for reliable prospective evaluation of serosal involvement as an adverse prognostic factor in GIST [34].

Indistinguishably, based on the macroscopic/histologic presence or absence of normal tissue between deeper tumor border and serosa, respectively, our case belongs to type II (extramural) high risk tumor, but without synchronous or metachronous metastases.

Less than 5% of GISTs are KIT-immunonegative; many of these tumors have activating mutations of *PDGFRA*, some of which are also inhibited by imatinib. It is becoming evident that alternative approaches to direct KIT inhibition will be required for long-term survival of patients with advanced GISTs [35].

Although rare, GISTs should be considered in the differential diagnoses of perforated gastrointestinal masses. Skipworth *et al*. presented the first English report of a perforated gastric GIST. Six further published reports describing the presentation of small bowel GISTs with perforation are reviewed [36].

We describe a rare case of small intestinal GIST presenting without perforation.

Boudabous *et al.* collected 24 cases of GIST (confirmed by the positivity of CD117 and/or CD33) analyzing demographic characteristics, clinical pattern, investigations treatment and therapeutic variables of patients. In 13 out of 24 cases the endoscopic appearance showed the tumor arising from muscular layer found in the stomach (54%), small bowel in four cases (16.5%) and duodenal or rectum in three patients (12.5%). The prognostic predictive factors identified were the size of tumor ≥10cm and the mitotic index [37].

Taking into account prognostic predictive factors found in our case, such as the size of the tumor 10cm, high mitotic index >5 per 50 HPF as well as extramural growth, there is a great chance of recurrence of the tumor.

Recent review articles focus on histopathologic criteria but omit clinical features and course of disease. In nonsyndromal CD117-positive GIST, girls tend to show more high-grade tumors and existing literature on pediatric GIST shows a 2.7-fold higher incidence in females. Altogether epithelioid cell tumors are most frequent, although in boys spindle cell tumors are reported more often. Together with known differences in molecular changes and local as well as systemic tumor behavior this strongly suggests that pediatric GIST represents a different entity from adult GIST [38].

Belev *et al.* and Menéndez *et al.* investigated primarily the prognostic value of Ki-67, as well as other parameters, in GISTs. Ki-67 presents a significant prognostic factor for GIST recurrence, which could be of great importance in evaluating the malignant potential of disease [39,40].

Our case shows a low proliferative index based on Ki-67 expression (10%). No significant association was noted between HR Fletcher score and Ki-67 score.

The standard therapy for GIST is complete surgical resection with safety margins of 1 to 2cm. Patients can achieve complete remission when thorough surgical resection is performed. Moreover, incomplete resection, including debulking surgery, does not seem to prolong survival [41].

Morrison and Hodgdon reported two cases of patients who were presented to an emergency department with signs and symptoms of small bowel obstruction. The pathologic diagnosis of small bowel GIST tumor was the same in both cases. Each tumor had a different method of obstruction, with one causing a volvulus and the other an intraluminal obstruction; however, both were successfully removed laparoscopically [42].

Likewise, our case clinically caused gastrointestinal hemorrhage and obstruction.

## Conclusions

GIST should be included in the differential diagnoses of intestinal mesenchymal tumors presenting as a single mass in young female adults. Obstruction from GIST tumors of the small bowel is a relatively rare occurrence, but should be considered in the differential diagnosis when other causes are not readily apparent and a solid lesion is demonstrated in the small bowel. Fletcher’s criteria are a useful prognostic classification because when applicable they are consistent with the evolution and prognosis of disease. Given that GIST in young adults (under 40 years of age) represents a more heterogeneous group, than GIST in pediatric cases, more effort should be made to investigate its pathogenesis and potentially more specific treatment.

## Consent

Written informed consent was obtained from the patient for publication of this case report and any accompanying images. A copy of the written consent is available for review by the Editor-in-Chief of this journal.

## Abbreviations

AURKA: Aurora kinase A; GIST: Gastrointestinal stromal tumor; HPF: High-power field; ICCs: Interstitial cells of Cajal; KIT: receptor tyrosine kinase encoded by proto-oncogene c-Kit; PDGFRA: Platelet-derived growth factor receptor alpha.

## Competing interests

The authors declare that they have no competing interests.

## Authors’ contributions

All of the authors were involved in the conception of the case report, the data collection and the literature review as well as in writing the manuscript. SMK performed gross and histological examination of resected specimen, including the immunohistochemistry interpretation and was a major contributor in writing the manuscript. IK and LK reviewed the literature. MK contributed to data collection and to histological and immunohistochemistry interpretation. VSM and AJS analyzed and interpreted the clinical data and were involved in drafting the manuscript. VK contributed in the selection of slides for illustration of the case. All authors read and approved the final manuscript.
